# BMPER Improves Vascular Remodeling and the Contractile Vascular SMC Phenotype

**DOI:** 10.3390/ijms24054950

**Published:** 2023-03-03

**Authors:** Franziska Pankratz, Aziza Maksudova, Roman Goesele, Lena Meier, Kora Proelss, Katia Marenne, Ann-Kathrin Thut, Gerhard Sengle, Annkatrin Correns, Jeanina Begelspacher, Deniz Alkis, Patrick M. Siegel, Christian Smolka, Sebastian Grundmann, Martin Moser, Qian Zhou, Jennifer S. Esser

**Affiliations:** 1 Department of Cardiology and Angiology, Heart Center Freiburg University, Faculty of Medicine, University of Freiburg, 79106 Freiburg, Germany; 2 Center for Biochemistry, Faculty of Medicine, University Hospital Cologne, University of Cologne, 50923 Cologne, Germany; 3 Department of Pediatrics and Adolescent Medicine, Faculty of Medicine, University Hospital Cologne, University of Cologne, 50923 Cologne, Germany; 4 Center for Molecular Medicine Cologne (CMMC), University of Cologne, 50923 Cologne, Germany; 5 Cologne Center for Musculoskeletal Biomechanics (CCMB), 50931 Cologne, Germany; 6 Institute of Anatomy, University of Veterinary Medicine Hannover Foundation, 30559 Hannover, Germany; 7 Department of Internal Medicine, University Hospital Basel, 4031 Basel, Switzerland

**Keywords:** growth factors/cytokines, remodeling, restenosis, vascular disease, BMPER, vSMCs, neointima

## Abstract

Dedifferentiated vascular smooth muscle cells (vSMCs) play an essential role in neointima formation, and we now aim to investigate the role of the bone morphogenetic protein (BMP) modulator BMPER (BMP endothelial cell precursor-derived regulator) in neointima formation. To assess BMPER expression in arterial restenosis, we used a mouse carotid ligation model with perivascular cuff placement. Overall BMPER expression after vessel injury was increased; however, expression in the tunica media was decreased compared to untreated control. Consistently, BMPER expression was decreased in proliferative, dedifferentiated vSMC in vitro. C57BL/6_Bmper+/− mice displayed increased neointima formation 21 days after carotid ligation and enhanced expression of Col3A1, MMP2, and MMP9. Silencing of BMPER increased the proliferation and migration capacity of primary vSMCs, as well as reduced contractibility and expression of contractile markers, whereas stimulation with recombinant BMPER protein had the opposite effect. Mechanistically, we showed that BMPER binds insulin-like growth factor-binding protein 4 (IGFBP4), resulting in the modulation of IGF signaling. Furthermore, perivascular application of recombinant BMPER protein prevented neointima formation and ECM deposition in C57BL/6N mice after carotid ligation. Our data demonstrate that BMPER stimulation causes a contractile vSMC phenotype and suggest that BMPER has the potential for a future therapeutic agent in occlusive cardiovascular diseases.

## 1. Introduction

Cardiovascular diseases (CVD) are by far the leading cause of death worldwide, with approximately 17.8 million CVD deaths in 2017 [1]. Myocardial infarction, stroke, or peripheral artery diseases are often caused by atherosclerotic lesions, which are one of the main reasons for vessel occlusion [2]. Percutaneous interventions such as balloon dilatation followed by stent implantation are a common therapeutic strategy. However, a common complication after these interventional procedures is neointimal hyperplasia and restenosis [3]. 

Under physiological conditions, vascular smooth muscle cells (vSMCs) are highly differentiated cells of the arterial tunica media that regulate the vascular tone. A specific set of genes of the contractile apparatus, such as smooth muscle actin (SMA), calponin (Calp), myosin heavy chain 11 (Myh11), or transgelin (TAGLN), is highly expressed in vSMCs and marks the differentiated, contractile phenotype. In response to vessel injury and mechanical stress, contractile vSMC can dedifferentiate, which allows them to proliferate, migrate, and produce the extracellular matrix (ECM). This synthetic myofibroblastic phenotype of vSMCs is found to accelerate atherosclerosis, hypertension, and neointima formation [4]. Therefore, a profound understanding of the molecular mechanisms that control the vSMC phenotype is necessary to develop preventive and targeted therapeutic strategies for vascular diseases [5]. Several growth factors are well known to modulate vSMC phenotypic plasticity: e.g., platelet-derived growth factor (PDGF) and insulin-like growth factor (IGF) induce the synthetic phenotype, whereas members of the transforming growth factor β (TGF-β) family shift the balance towards a contractile phenotype [6].

In recent years, it has become evident that the TGF-β superfamily plays an important role during vascular development, homeostasis, and remodeling [7]. The bone morphogenetic proteins (BMPs) are the largest subgroup of these extracellular proteins that signal through cell-surface complexes of type I and type II serine/threonine kinase receptors. The components of the BMP signaling pathway, i.e., the ligands, receptors, and intracellular signaling molecules, are all expressed in vascular cells [8]. Upon activation, the receptors mediate intracellular signaling via the classical Smad 1/5 transcription factor phosphorylation and by alternative pathways such as MAP kinases and phosphoinositide 3-kinase (PI3K) pathways [9]. Recently, BMPs have been implicated as important regulators of vSMC plasticity. Several studies have reported BMP signaling to modulate the differentiation of vSMCs by inhibiting proliferation and migration and to promote vSMC differentiation into a contractile phenotype [10,11]. 

The BMP signaling pathway is highly regulated at several levels. For example, BMP receptor availability is regulated at the cell membrane by co-receptors, pseudo-receptors, and proteases [12,13]. In the extracellular space, the availability of BMPs is regulated by BMP antagonists such as chordin [14], noggin [15], and BMP modulators such as twisted gastrulation (TSG) [16] and the BMP endothelial cell precursor-derived regulator (BMPER) [17]. BMPER, also known as the vertebrate homolog of Drosophila cross-veinless 2, is a secreted glycoprotein that contains five cysteine-rich domains followed by a von Willebrand D domain and a trypsin inhibitor domain. It was originally identified in a screening for differentially expressed proteins in embryonic endothelial precursor cells [17]. We and others have previously shown that BMPER may enhance BMP signaling in a concentration-dependent fashion [18,19,20]. BMPER is able to bind to BMP-2, -4, -6, -7, -9, and BMPRIa/b [17,19,21]. Lately, BMPER has been the subject of intensive research in the area of vascular biology including coronary artery development [22], inflammation [23,24], atherosclerosis [25], and angiogenesis [18,26]. However, the focus of all prior investigations was on endothelial cells, and little is known regarding the role of BMPER in vSMCs biology. In earlier investigations, BMPER expression during embryonic development was detected in the somites and in migrating neural crest cells at E9.5. Later on, BMPER is expressed in the aorto–gonad–mesonephros (AGM) region that gives rise to hemangioblast cells and, therewith, vascular development [17,27]. Of interest, vSMCs have been reported to originate from all these sources [28], suggesting that BMPER might play a role in vSMC biology. Therefore, we aimed to investigate the function of BMPER in a mouse model of neointima formation in the carotid artery and in vSMCs phenotype modulation. 

## 2. Results

### 2.1. BMPER Expression in Healthy and Injured Carotid Artery

**Figure 1:**
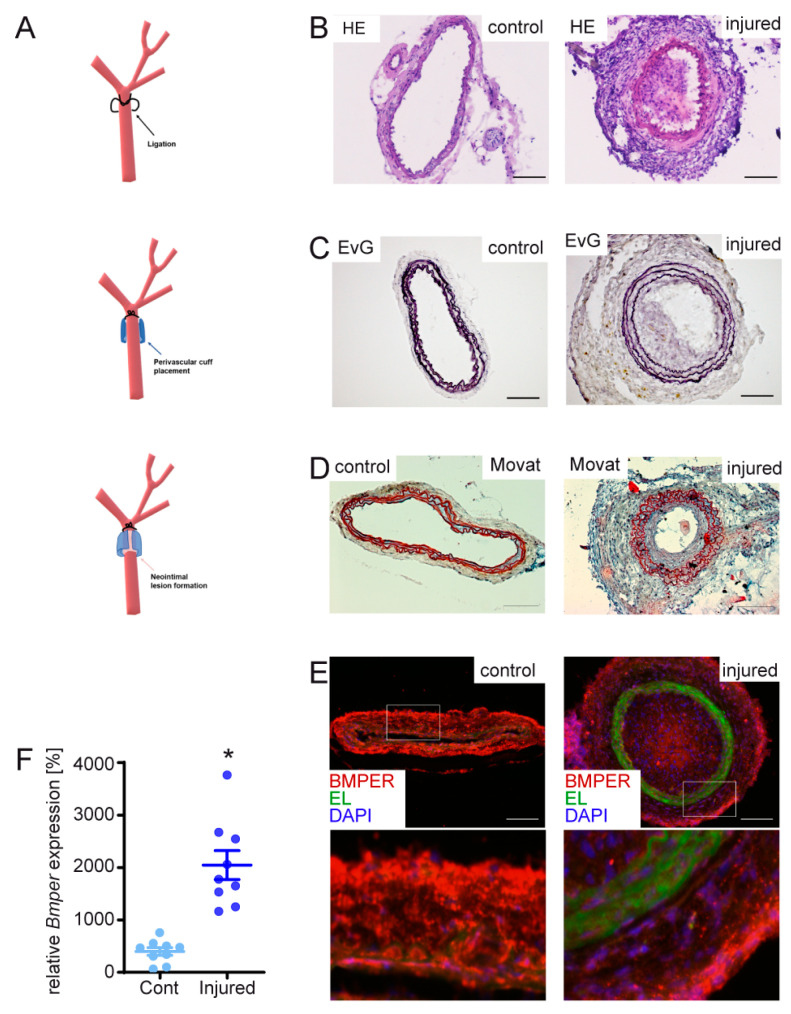
Mouse carotid artery ligation and cuff-induced neointimal formation model causes changes in bone morphogenetic protein-binding endothelial regulator (Bmper) expression in the carotid artery vessel wall. (**A**) Scheme of carotid injury mouse model. In 10*–*12-week-old C57BL/6N male mice, the right common carotid artery (RCCA) was dissected and ligated distal to the carotid bifurcation, and a perivascular polyethylene cuff was placed around the vessel. For histology analysis of tissue sections, RCCA and LCCA were harvested after 21 days and frozen in embedding media before cryosections were prepared. (**B**) Representative micrographs of sections from the LCCA (**left**, untreated) and RCCA (**right**, injured) stained with hematoxylin and eosin (HE). (**C**) Elastic van Gieson (EvG) and (**D**) Movat pentachrome stain. (**E**) Representative micrographs of BMPER immunostaining (red), elastic laminae (EL, green autofluorescence), and 4′,6-diamidino-2-phenylindole (DAPI) to visualize nuclei (blue) with a 200× magnification of the indicated rectangle. Scale bar: 100 μm. (**F**) After 14 days, RCCA and left CCA (LCCA) were harvested and RNA was isolated. Quantitative real-time PCR analysis of *Bmper* mRNA expression levels 14 days after carotid injury in C57BL/6N mice. RNA polymerase II serves as internal control. Data represent mean values with SEM; (**A**), *n* = 9 per group; * *p* = as indicated vs. control.

Previously, we and others have shown that BMPER is expressed in endothelial cells and in the aortic wall [18,21]. However, to the best of our knowledge, the expression of BMPER in vascular disease has not been reported yet. To investigate BMPER expression in pathological arterial blood vessel remodeling such as neointima formation, we performed a mouse carotid ligation model combined with perivascular cuff placement to induce restenosis of the right common carotid artery (RCCA) (Figure 1A). With this approach, we aimed to focus on the role of vSMCs during arterial remodeling compared to other models such as a wire injury. Complete ligation of the artery distal to the carotid bifurcation induces rapid proliferation of medial SMCs, leading to extensive neointima formation while the endothelial cell layer is not disturbed [29]. In addition, cuff placement further increases mechanical stress and inflammation of the adventitial layer [30]. Moreover, the cuff allows the application of hydrogel for local substance delivery. As a simple approach to test if *Bmper* expression is regulated during neointima formation, we isolated RNA after 14 days from the RCCA and the left CCA (LCCA) and performed qPCR analysis. *Bmper* mRNA expression was increased after carotid injury compared to the untreated LCCA (Figure 1F). For histological analysis mice were sacrificed, and the RCCA as well as the untreated LCCA were isolated after 21 days. To investigate neointima formation in our carotid injury model’s hematoxylin and eosin (Figure 1B), Elastic van Gieson (Figure 1C) and Movat’s stain (Figure 1D) were performed. Immunostaining showed that Bmper was expressed in endothelial cells and vSMCs in LCCA. Interestingly, Bmper expression decreased in the intimal and medial layers in the RCCA after carotid injury. In contrast, Bmper expression strongly increased in the adventitia (Figure 1E), which is in line with the finding on the mRNA expression level. Taken together, Bmper expression is differentially regulated in the carotid artery wall after injury, supporting the notion that it may play a role during neointima formation.

### 2.2. Loss of Bmper in C57BL/6N Mice Exacerbates Intimal Hyperplasia and ECM Remodeling 

**Figure 2:**
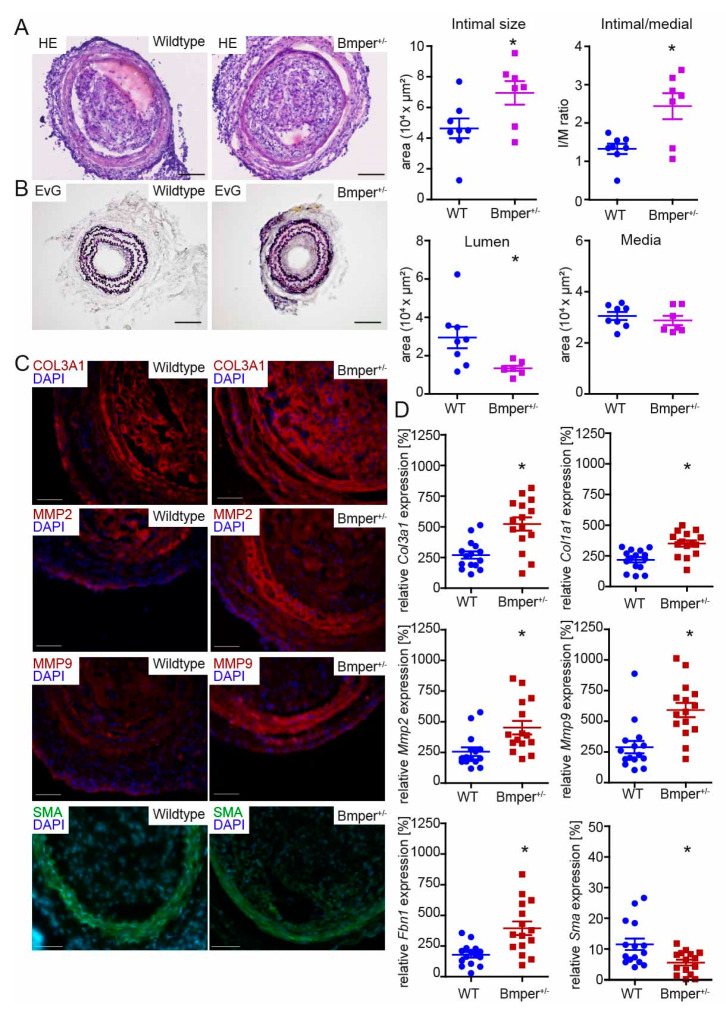
Bmper-deficiency in C57BL/6N mice exacerbates intimal hyperplasia and extracellular matrix (ECM) deposition after injury. (**A**) Representative HE and (**B**) EvG stain of carotid artery tissue sections from wildtype littermate or *Bmper*+/− mice 21 days after carotid injury are shown with quantification of intimal size and intimal/media ratio, lumen, and media size. Scale bar: 100 μm. (**C**) Representative photomicrographs of cross-sections with immunostaining against collagen type III alpha 1 chain (Col3a1/red), matrix metalloproteinase 2 (Mmp2/red), Mmp9 (red), and smooth muscle actin (Sma/green). DAPI stain to visualize nuclei (blue). Scale bar: 50 μm. Data are expressed as mean ± SEM; *n* = 8 wildtype, *n* = 7 *Bmper*+/−; * *p* = as indicated. Controls are available in Figure S1C. (**D**) Quantitative real-time PCR analysis of *Col3a1*, *Col1a1*, *Mmp2*, *Mmp9*, *fibrillin 1 (Fbn1)* and *Sma* mRNA expression levels 14 days after carotid injury. RNA polymerase II serves as internal control. Gene expression is displayed relative to untreated LCCA of wildtype mice. Data represent mean ± SEM; *n* = 15 per group; * *p* = as indicated.

Given that Bmper expression is increased during neointima formation, we aimed to further investigate the function of Bmper in vivo. Because *Bmper* homozygous-deficient mice die at birth [20], we used BMPER heterozygous-deficient (*Bmper*+/−) mice to investigate the effect of Bmper deficiency after vascular injury. Three weeks post carotid injury, mice were sacrificed and carotid arteries were subjected to histopathological examination. Hematoxylin and eosin staining revealed increased stenosis (Figure 2A) in *Bmper*+/− mice and Elastic van Gieson staining (Figure 2B) demonstrated an increase in neointimal formation compared to wildtype littermates. Quantification of intimal size, media, and lumen revealed a significant increase in intimal/medial ratio in *Bmper*+/− mice along with reduced vessel lumen compared to littermates. Dedifferentiation of vSMCs is associated with increased ECM deposition, including deposition of collagens, increased MMP expression to facilitate proliferation and migration, and reduced contractile marker gene expression [31]. Therefore, collagen 3 type A1 (Col3a1), Mmp2, Mmp9, and Sma expression were further examined by immunohistological analysis 3 weeks after carotid ligation. In *Bmper*+/− mice, Col3a1 was increased in the arterial wall, especially in the adventitia, compared to wildtype mice (Figure 2C). Interestingly, Mmp2 and Mmp9 were clearly and especially increased in the medial layer of *Bmper*+/− mice compared to littermates (Figure 2C). Accordingly, Sma expression was reduced in *Bmper*+/− mice (Figure 2C). We corroborated and extended our findings by analyzing mRNA expression levels of *Col3a1*, *Col1a1*, *Mmp2*, *Mmp9*, *fibrillin-1 (Fbn1)*, and *Sma* two weeks after carotid ligation (Figure 2D). Furthermore, as the transgene *lacZ* is integrated into the genomic *Bmper* gene locus as reporter [32], we used *lacZ* transgene expression to corroborate our findings from wildtype C57BL/6N mice that showed increased Bmper mRNA expression in neointima formation (Figure S1). Taken together, Bmper-deficiency resulted in enhanced expression of ECM components accompanied by increased Mmp2 and Mmp9 and reduced contractile marker Sma expression. These findings are in line with the enhanced neointima formation following injury in *Bmper*+/− mice.

### 2.3. BMPER-Silencing in Human vSMCs Leads to a Synthetic vSMC Phenotype

**Figure 3:**
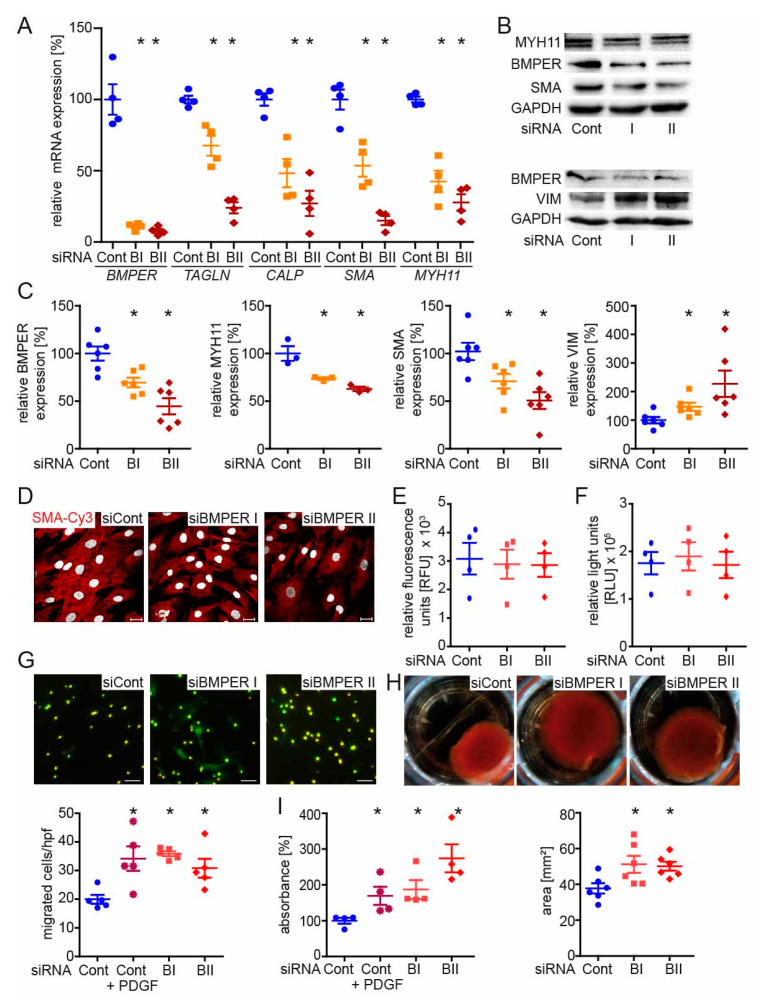
BMPER silencing in human primary vascular smooth muscle cells (vSMCs) leads to a synthetic SMC phenotype. Human vSMCs were silenced for BMPER with either of two specific small interfering RNAs (siRNAs; BI and BII) or transfected with control siRNA (Cont). (**A**) Quantitative real-time PCR analysis of *BMPER*, *transgelin (TAGLN)*, *calponin (CALP)*, *SMA*, and *myosin heavy chain 11 (MYH11)* mRNA expression levels 72 h post-transfection. Human RNA polymerase II serves as internal control. Data are mean ± SEM; *n* = 4; * *p* < 0.0045 vs. control. (**B**,**C**) Western blot analysis of MYH11, BMPER, SMA, and vimentin (VIM) protein expression was performed 72 h post siRNA transfection. Glyceraldehyde 3-phosphate dehydrogenase (GAPDH) serves as loading control. Data represent mean ± SEM; *n* = 6; * *p* < 0.0281 vs. control. (**D**) Representative photomicrographs of SMA (red) in human vSMCs 72 h post-transfection and 48 h after stimulation with BMP4 (c = 50 ng/mL) to show contractile fibers. Nuclei were stained with DAPI (white). Scale bars: 20 μm. (**E**) Cell viability (CellTiter-Fluor™) and (**F**) cell apoptosis (Caspase-Glo*^®^*3/7) assays were performed 48 h after siRNA transfection. RFU: relative fluorescence units. RLU: relative light units. Data are mean ± SEM; *n* = 3. (**G**) Transmigration assay was performed 48 h post-transfection. Depicted are representative photomicrographs with the pores in bright light and vSMCs in green (live-cell tracker). Below quantification is shown. Platelet-derived growth factor (PDGF c = 40 ng/mL) served as a positive control for increased vSMC migration. Scale bars: 20 μm. hpf indicates high-power field. Data represent mean ± SEM; *n* = 5; * *p* < 0.01731 vs. control. (**H**) Representative images of collagen-gel cell contraction assay and quantification (below). Six hours post-siRNA transfection vSMCs are seeded in a collagen gel and incubated for 48 h before gels were released for contraction. Data are mean ± SEM; *n* = 6; * *p* < 0.0352 vs. control. (**I**) Proliferation was determined by BrdU assay 48 h post-siRNA transfection. Data represent mean ± SEM; *n* = 4; * *p* < 0.0393 vs. control.

In order to investigate the impact of BMPER deficiency on vSMC phenotype and function, human vSMCs were silenced for BMPER with either of two specific small interfering RNAs (siRNAs) or transfected with scrambled siRNA as control. First of all, we assessed the efficient *BMPER* knockdown in a time course of 24*–*72 h on mRNA level by PCR followed by an analysis of contractile marker mRNA expression such as *TAGLN*, *CALP*, *SMA*, and *MYH11* (Figure 3A). While BMPER expression was reduced, contractile marker expression was decreased (Figure 3A–C). On the other hand, expression of the synthetic vSMC marker vimentin was increased in BMPER-deficient cells (Figure 3B,C). However, not only was the expression of contractile marker SMA decreased in BMPER-silenced vSMCs but also the assembly of the contractile apparatus was diminished compared to siRNA control (Figure 3D). Changes in cell viability or apoptosis by BMPER silencing were excluded (Figure 3E,F). As a synthetic vSMC phenotype is associated with changes in cell function, we next investigated vSMC migration, contraction, and proliferation (Figure 3G–I). Indeed, cell migration and proliferation were increased 48 h after *BMPER* siRNA transfection, and collagen gel contraction was reduced. Collectively, we found that silencing of BMPER in vSMCs led to decreased contractile and increased synthetic marker expression, along with increased proliferation and migration, as well as decreased gel contraction emphasizing a synthetic vSMC phenotype.

### 2.4. Recombinant BMPER Protein Stimulation of Human vSMC Induces a Contractile Phenotype

**Figure 4:**
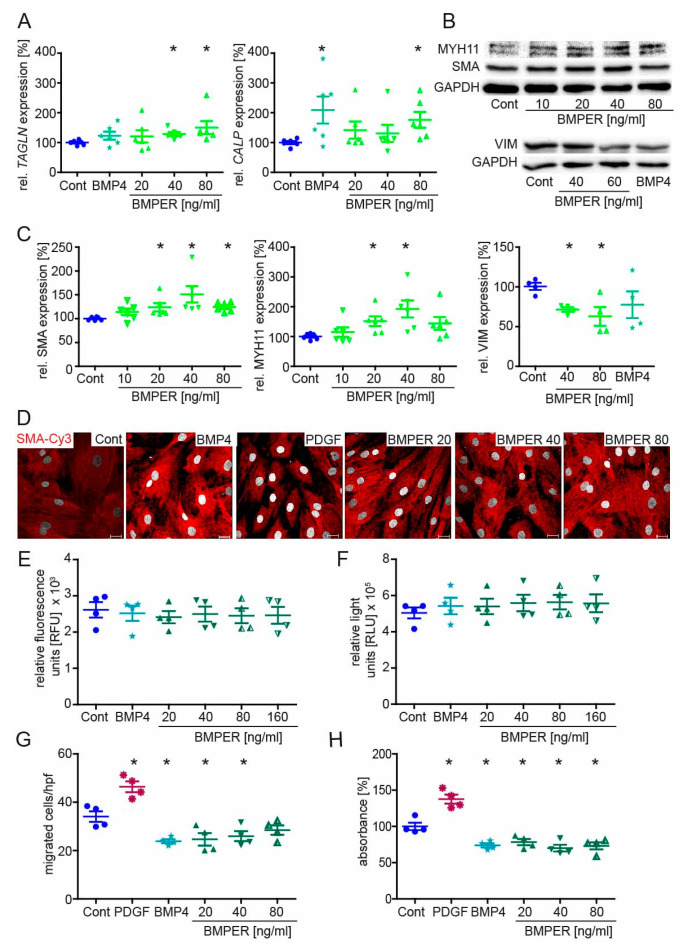
Recombinant BMPER protein stimulation of human vSMC induces a contractile phenotype. After initial serum starvation in 0.4% EBM for 24 h, vSMCs were stimulated with recombinant BMP4 (c = 40 ng/mL) or indicated concentrations of BMPER protein for 48*–*72 h before expression analysis of contractile markers or cell culture assays were performed. (**A**) Quantitative realtime-PCR analysis of *TAGLN* and *CALP* mRNA. Human RNA polymerase II serves as internal control. Data are mean ± SEM; *n* = 6; * *p* < 0.048 vs. control. (**B**) Expression of MYH11, SMA, and VIM protein in vSMCs stimulated with indicated BMPER concentrations or control. GAPDH serves as loading control. (**C**) Quantification of Western blot analysis. Data are mean ± SEM; *n* = 6 (MYH11, SMA) *n* = 4 (VIM); * *p* < 0.024 vs. control. (**D**) Vascular SMCs stimulated for 72 h with BMP4 (c = 50 ng/mL), PDGF (c = 20 ng/mL), or different concentrations of BMPER were subjected to immunofluorescence staining for SMA (red) to detect contractile fiber formation. Nuclei were stained with DAPI (white). Scale bar, 20 µm. (**E**) Cell viability and (**F**) cell apoptosis assays. Data are mean ± SEM; *n* = 4. (**G**) Transmigration assay was performed after 48 h of stimulation. PDGF serves as positive control for increased vSMC migration. Data represent mean ± SEM; *n* = 4; * *p* < 0.0367 vs. control. (**H**) Proliferation was determined by BrdU assay. Data represent mean ± SEM; *n* = 4; * *p* < 0.0176 vs. control.

Given that the silencing of BMPER promotes the synthetic vSMC phenotype, we next asked if stimulation with recombinant human BMPER protein had an inverse effect on the vSMC phenotype. Before stimulation with different concentrations of BMPER protein, vSMCs were put on starvation medium for 24 h. Stimulation with BMP4 protein served as positive control to induce contractile markers [10]. As expected, BMPER stimulation of vSMCs increased *TAGLN*, *CALP* (Figure 4A), and *SMA* (Figure S2A) mRNA expression. In addition, MYH11 and SMA protein expression was enhanced and, conversely, VIM protein expression was reduced after BMPER stimulation (Figure 4B,C). After BMP4 and BMPER stimulation, immunocytochemistry staining of SMA showed increased contractile filaments in vSMCs compared to unstimulated control, particularly compared to vSMCs stimulated with PDGF, which is known to evoke a synthetic vSMC phenotype (Figure 4D). Cell viability and apoptosis assays revealed no effect of BMP4 or BMPER on vSMCs (Figure 4E,F). BMP4 and BMPER protein stimulation reduced vSMC migration and proliferation in contrast to PDGF (Figure 4G,H). In summary, we confirmed our notion that recombinant BMPER promotes a contractile phenotype in vSMCs in vitro. 

### 2.5. BMPER and BMP4 Expression Are Reduced in Serum-Stimulated Synthetic vSMCs

**Figure 5:**
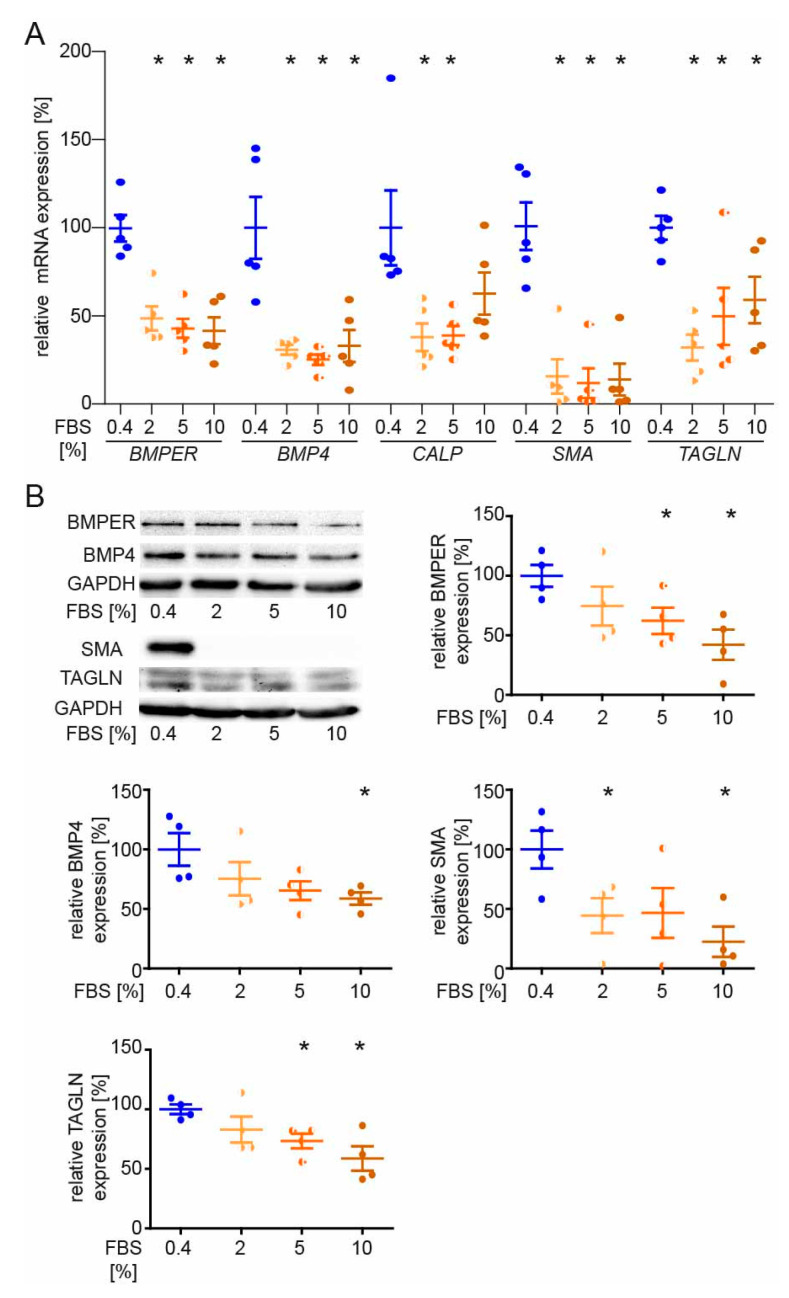
BMPER and BMP4 expression are reduced in serum-stimulated synthetic vSMCs. After initial serum starvation in 0.4% EBM for 24 h, vSMCs were stimulated with indicated concentrations of FBS for 48 h before expression analysis of contractile markers was performed. (**A**) Quantitative realtime-PCR analysis of *BMPER*, *BMP4*, *CALP*, *SMA*, and *TAGLN* mRNA. Human RNA polymerase II serves as internal control. Data are mean ± SEM; *n* = 5; * *p* < 0.0256 vs. control. (**B**) Western blot analysis of BMPER, BMP4, SMA, and TAGLN protein expression was performed at 48 h of FBS stimulation. GAPDH serves as loading control. Data represent mean ± SEM; *n* = 4; * *p* < 0.0426 vs. control.

As BMPER shifts the vSMC phenotype towards a contractile phenotype, we hypothesized that the expression of BMPER and BMP4 is downregulated in vSMCs that adopt a synthetic phenotype. Therefore, serum-starved vSMCs were stimulated with increasing concentrations of FBS that is commonly used to stimulate SMC proliferation and dedifferentiation in vitro [33]. Diminished expression of the contractile markers *SMA* and *TAGLN* were used as positive controls (Figure 5A), indicating that vSMCs had switched towards the synthetic phenotype. Along this line, *BMPER* and *BMP4* expression is downregulated. In addition, expression of BMPER, BMP4, SMA, and TAGLN in vSMCs after FBS stimulation was further confirmed on the protein level (Figure 5B). To investigate if the classical SMAD signaling cascade is affected by decreased BMPER expression, we silenced BMPER in vSMCs and performed Western blot analysis 48 h post-siRNA transfection (Figure S2B). Similar to our previous findings in endothelial cells that reduced BMPER expression decreases SMAD signaling [18], we now confirmed this finding in vSMCs. Taken together, BMPER and BMP4 along with contractile markers are downregulated in vSMCs that switch to a synthetic phenotype.

### 2.6. BMPER Physically Interacts with Insulin-like Growth Factor-Binding Protein 4 (IGFBP4)

**Figure 6:**
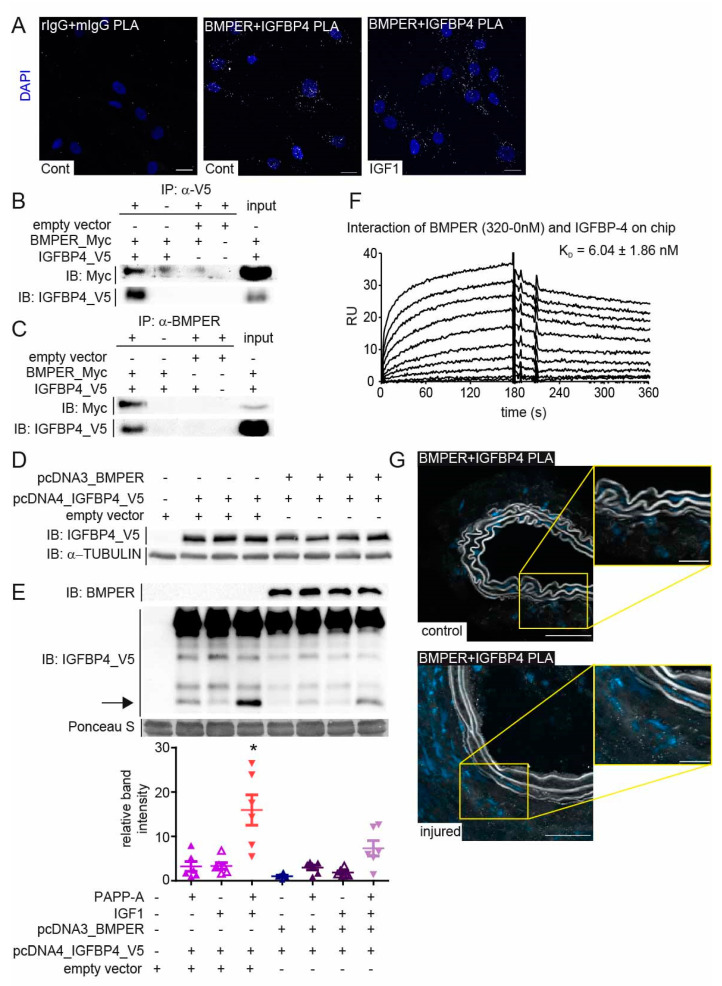
BMPER physically interacts with insulin-like growth factor-binding protein 4 (IGFBP4). (**A**) Representative photomicrographs of in situ proximity ligation assay (PLA) showing the interaction between BMPER and IGFBP4. Human vSMCs were either incubated in 0.4% FBS/EBM or treated with IGF1 (c = 10 ng/mL) for 20 min before the association of BMPER and IGFBP4 was visualized using an in situ PLA (white). As control, respective rabbit and mouse primary IgGs were used to assess unspecific antibody interactions. Nuclei were stained with DAPI (blue). Scale bars: 20 µm. (**B**,**C**) HEK293A cells were transfected with BMPER-Myc and IGFBP4-V5 or empty vector for 24 h. (**B**) Immunoprecipitation (IP) of V5-tagged IGFBP4 using a V5-specific antibody was performed. (**C**) BMPER was immunoprecipitated with a BMPER-specific antibody from HEK293A cell lysates 24 h post-transfection. Normal IgG antibody served as IP control (-). Input represents lysate not subjected to IPs. IPs were repeated at least 3 times. (**D**,**E**) BMPER and IGFBP4 protease activity assay. (**D**) Twenty-four hours after plasmid transfection in HEK293A cells, lysates were subjected to Western blot analysis to verify equal overexpression of IGFBP4 between samples. TUBULIN served as loading control. (**E**) Cell-free supernatants were preincubated with IGF1 (c = 25 ng/mL) as indicated before PAPP-A (c = 150 ng/mL) was added. Afterwards, supernatants were concentrated and subjected to Western blot analysis. Arrow indicates cleaved IGFBP4 at approximately 14 kDa, which is quantified in the diagram below. Ponceau S stain served as loading control. Data are mean values ±SEM; *n* = 6; One-way ANOVA; * *p* < 0.001 vs. all other conditions. (**F**) Representative surface plasmon resonance (SPR) sensorgram showing the interaction of immobilized IGFBP-4 with soluble BMPER (KD of 6.04 ± 1.86 nM). The 0*–*320 nM BMPER diluted in HBS-EP buffer was injected for 180 s (injection stop) followed by a dissociation for another 180 s. (**G**), Representative BMPER and IGFBP4 PLA photomicrographs of RCCA and LCCA 14 days after carotid ligation. Association of BMPER and IGFBP4 in white dots and nuclei in blue (DAPI). Scale bars: 20 µm.

Many extracellular proteins such as the matricellular protein cellular communication network factor (CCN2) mediate pleiotropic effects by binding to different growth factors or other ECM components [34]. We and others have shown that BMPER binds and regulates the function of BMP2, 4, 6, 7, and 9 [35]. However, if we assume that BMPER in high concentrations inhibits BMP4 and both BMPER and BMP4 can induce a contractile phenotype in vSMCs, BMPER seems to act in an additional way to promote the contractile phenotype. In yeast two-hybrid screening performed in the past, we identified that IGFBP4 physically interacts with BMPER. To confirm our findings, we performed a PLA with BMPER and IGFBP4 antibodies to show a physical interaction in vSMCs. The PLA of serum-starved vSMCs with species-specific control antibodies shows only a few unspecific signals compared to PLA with BMPER and IGFBP4-specific antibodies (Figure 6A). Moreover, in order to verify specific fluorescence signals, we stimulated the vSMCs for 20 min with IGF1 to activate the IGF pathway. Indeed, changes in the fluorescent signals in the cellular localization were detectable compared to serum-starved vSMCs. To further confirm a physical BMPER–IGFBP4 interaction, we performed co-immunoprecipitation (IP) assays in HEK293A. Cells were transfected with BMPER-Myc and IGFBP4-V5 tagged or empty control vectors for 24 h before IP, using V5-specific or BMPER-specific antibody, as shown in Figure 6B,C, respectively, was performed. To corroborate our findings and to gain better insight into the interaction of BMPER with IGFBP4 and its potential impact on BMP4 binding, we employed surface plasmon resonance (SPR)-binding studies. When increased concentrations of BMPER were flown over immobilized IGFBP4 on a sensor chip, robust binding signals were detected, as shown by association curves after injection and dissociation curves after injection stop (Figure 6F). By assessing the kinetic parameters of both curves, a high affinity binding constant of K_D_ = 6 nM was determined for this interaction. As shown previously, soluble BMPER flown over immobilized BMP4 also showed a high binding affinity of 1.5 nM (Figure S3A). However, in contrast, when IGFBP4 was flown over immobilized BMP4, no interaction was detected (Figure S3B). To assess whether the presence of IGFBP4 affects the interaction of BMPER with BMP4, a stable concentration of BMPER was flown over immobilized BMP4 in the presence of increased concentrations of IGFBP4. Our results from this competition assay revealed that IGFBP4 does not interfere with the BMPER–BMP-4 interaction (Figure S3C).

While BMPER and BMP4 expression was decreased in vSMCs after serum stimulation (Figure 5), IGFPB4 expression was not significantly downregulated in serum-stimulated vSMCs (Figure S4A). The function of IGFBP4 is to bind and inhibit IGF binding its receptor in the extracellular space. IGFBPs are in turn post-translationally modulated by specific proteases that release IGF. In vSMC, activation of the IGF signaling pathway is associated with increased migration and proliferation, as well as neointima formation [36]. Along this line, siRNA-mediated silencing of IGFBP4 in vSMCs decreased the expression of the contractile markers *TAGLN*, *CALP*, and *SMA* (Figure S4B). As we have shown that BMPER binds to IGFBP4, we next asked if BMPER protects IGFBP4 from cleavage by pregnancy-associated plasma protein-A (PAPP-A) and thus inhibits the release of IGF, which could have a protective role in neointima formation. Again, we used HEK293A cells and overexpressed IGFBP4 and BMPER under serum-free conditions. We confirmed uniform overexpression of IGFBP4 in HEK293A lysates 24 h post transfection (Figure 6D). The supernatants containing the extracellular proteins IGFBP4 and BMPER were used in a cell-free assay to determine the relative contribution of PAPP-A to the total proteolysis of IGFBP-4 with and without the presence of BMPER and IGF1, which enhances proteolytic cleavage of IGFBP4 (Figure 6E). As reported, IGF1 allowed proteolytic cleavage of IGFBP4 by PAPP-A [37]. However, if BMPER is co-expressed with IGFBP4, IGF1-induced proteolytic cleavage by PAPP-A is significantly reduced. In situ analysis of *Bmper* and *Igfbp4* mRNA expression in injured and control carotid artery using RNAscope technology showed a similar expression pattern (Figure S4C) and confirmed the results of the immunohistofluorescence Bmper (Figure 1E) and lacZ stain (Figure S1B). Moreover, PLA of injured and control carotid artery showed a physical association in situ that is clearly enhanced in the adventitia of the injured carotid artery (Figure 6G). Taken together, these data demonstrate that BMPER, in addition to the BMPs, also interacts with the IGF signaling pathway regulator IGFBP4 and protects IGFBP4 against proteolytic cleavage. In turn, the release of IGF1 was diminished, suggesting that in this way the contractile vSMC phenotype can be preserved.

### 2.7. Recombinant BMPER Protein Prevents Intimal Hyperplasia and ECM Deposition in C57BL/6N Mice after Carotid Injury

**Figure 7:**
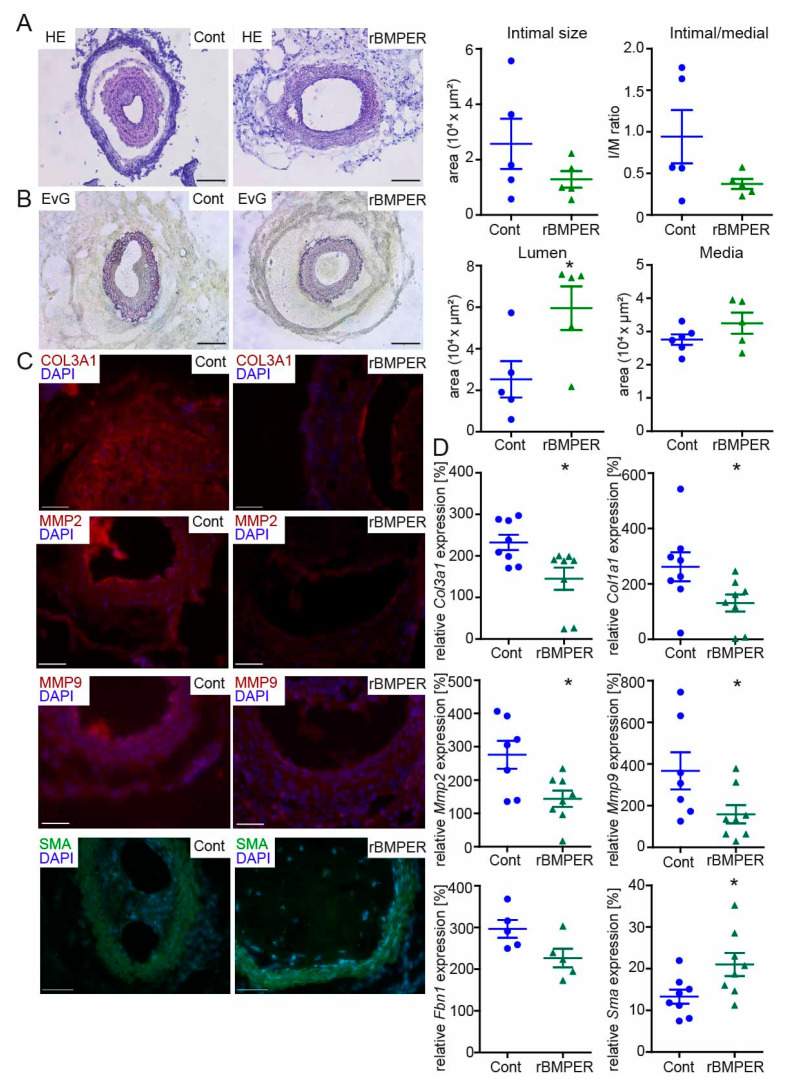
Recombinant Bmper protein prevents intimal hyperplasia and ECM deposition in C57BL/6N mice after carotid injury. In 10*–*12-week old male mice, the right common carotid artery (RCCA) was dissected and ligated distal the carotid bifurcation, and a perivascular polyethylene cuff was placed around the vessel. Thirty-percent Pluronic gel was applied to the cuff containing either mouse recombinant Bmper protein (c = 1 µg/mL) or solvent control. (**A**) Representative HE and (**B**) EvG stain of carotid artery tissue sections from mice with Pluronic gel and solvent control (**left**) compared to recombinant Bmper protein (**right**) 21 days after carotid injury are shown with quantification of intimal size, intimal/media ratio, lumen and media size. Scale bar: 100 μm. (**C**) Representative photomicrographs of cross-sections with immunostaining against Col3a1 (red), Mmp2 (red), Mmp9 (red), and Sma (green). DAPI stain to visualize nuclei (blue). Scale bar: 50 μm. Data are expressed as mean ± SEM; *n* = 5 per group; * *p* = as indicated. Controls are available in Figure S5. (**D**) Quantitative real-time PCR analysis of *Col3a1*, *Col1a1*, *Mmp2*, *Mmp9*, *Fbn1*, and *Sma* mRNA expression levels 14 days after carotid injury and Pluronic gel application. RNA polymerase II serves as internal control. Gene expression is displayed relative to untreated LCCA of wildtype mice. Data represent mean ± SEM; *n* = 8 per group, *Fbn1 n* = 5/group; * *p* = as indicated.

The therapeutic goal in the treatment of occlusive vessel disease is to prevent restenosis. Previously, we and others have found that BMPER exerts a moderate pro-angiogenic and anti-inflammatory effect on endothelial cells [18,20,23,24,38]. Combined with our novel findings that BMPER supports the contractile vSMC phenotype, BMPER appears to be an attractive candidate for a therapeutic approach in arterial vessel disease. To test this hypothesis, recombinant mouse BMPER (rBmper) protein was mixed in Pluronic gel, which in its sol state acts as a drug-delivery system in a physiological environment [39]. Carotid ligation, in combination with perivascular cuff placement, was used to induce restenosis, and the cuff allowed the local application of Pluronic gel. Three weeks post carotid ligation, together with Pluronic gel application, C56BL/6N mice were sacrificed and carotid arteries were subjected to histopathological examination. As expected, HE staining (Figure 7A) and EvG staining (Figure 7B) of mice treated with solvent control showed clear induction of neointima formation. However, local administration of rBmper reduced the neointimal area, which is indicative of diminished vSMC migration and proliferation. Quantification of intimal size, media, and lumen revealed in total a clear trend towards decreased intimal/medial ratio in rBmper mice along with significantly increased vessel lumen. In order to figure out the vSMC phenotype state, we next investigated changes in the expression of ECM composition and vSMC markers. Immunohistochemistry analysis showed markedly diminished Col3a1, Mmp2, and Mmp9 expression 3 weeks after carotid ligation in rBmper mice compared to solvent control mice (Figure 7C). Additionally, Sma expression was enhanced in the media of rBmper, overall indicating a more contractile phenotype of vSMCs. These results are corroborated and extended by analysis of mRNA expression levels of *Col3a1*, *Col1a1*, *Mmp2*, *Mmp9*, *Fbn1*, and *Sma* two weeks after carotid ligation (Figure 7D). Altogether, these data demonstrate that administration of rBmper in Pluronic gel during the process of restenosis in mice has the capacity to keep vSMCs in the contractile phenotype and, thus, reduce neointima formation.

## 3. Discussion

Unlike other muscle cells such as cardiomyocytes, vSMCs are not terminally differentiated. In response to injury or mechanical stress, contractile vSMCs change phenotype, proliferate, and migrate as part of the remodeling process. Dysregulation of this plasticity program is the reason for neointimal hyperplasia, which is the major cause for restenosis after percutaneous interventions [40]. Here, we report that after vessel injury, the expression of ECM protein Bmper is altered during the course of neointima formation. Both loss-of-function and gain-of-function studies suggest that Bmper plays a key role in vSMCs’ phenotypic switch in vitro and in vivo and that, presumably, in this way, Bmper limits neointima formation. Besides activating the BMP pathway, we revealed IGFBP4 as a new physical interaction partner for BMPER. This finding highlights a second way through which BMPER inhibits the IGF pathway and, thus, may promote the contractile vSMC phenotype.

Besides vSMC migration and proliferation in the subintimal space, a hallmark of neointima formation is the deposition of high amounts of ECM [41]. Interestingly, *Bmper* mRNA expression levels of lysates from injured carotid arteries compared to untreated arteries are upregulated after carotid ligation at a time point that is correlated with high ECM deposition. By now, it is well-accepted that there is a tight interplay between the ECM and the BMP signaling pathway [42], which would support the notion that Bmper is available and, thus, its expression is increased. However, examination of the immunohistological Bmper staining in untreated carotid artery displayed a good Bmper signal in the media, which 21 days after carotid ligation was lost, and Bmper expression was detected more in the adventitial tissue. In line with these findings, BMPER and BMP4 expression was reduced in cultured human vSMCs that were stimulated with FBS to promote SMC proliferation and de-differentiation. Further reasons for changes in BMPER expression could be changes in mechanics, e.g., blood flow, and BMP signaling feedback loops. Corriere et al. have reported increased Bmp4 expression after carotid ligation during neointima formation and assumed that endothelial Bmp4 expression is induced by alterations in blood flow [43]. Along the same lines, another study has shown that in response to disturbed flow conditions, BMP4 and BMP antagonists such as noggin are coexpressed in the arterial wall of mouse and human blood vessels [44]. Moreover, the authors hypothesize that the expression of antagonists seemed to play a negative feedback role against the inflammatory response of BMP4. In fact, signaling pathway autoregulation is a common theme for the BMP pathway and has been reported by several groups in different tissue contexts [21,45,46]. 

Our findings show that BMPER alongside the contractile markers is downregulated in vSMCs that adopt a synthetic phenotype. Moreover, our in vitro data demonstrate that BMPER deficiency triggers vSMC dedifferentiation, proliferation, and migration, supporting the role of BMPER in additionally regulating vSMC phenotypic switching. Accordingly, neointima formation in *Bmper* heterozygote-deficient mice is aggravated, and this supports the notion that in vivo reduced Bmper expression also increases the synthetic vSMC phenotype as the mouse in vivo model of restenosis used is predominantly a model for investigating the contribution of vSMCs to neointima formation [29,30]. This is in good agreement with several in vitro studies that have reported that BMP signaling influences the differentiation of vSMCs by inhibiting proliferation and migration and promoting vSMC differentiation into a contractile phenotype [11]. For example, Lagna et al. have reported that BMP2, BMP4, and BMP7 displayed a similar preserving effect on contractile vSMC-specific gene expression in different types of vSMCs, which is SMAD-dependent [10]. Moreover, the same group revealed that SMAD transcription factors regulate postranscriptional miRNA biogenesis that is critical for the control of the contractile vSMC phenotype [6]. In accordance with these findings, Corriere et al. reported that, besides increased Bmp4 expression after carotid ligation, constitutively active BMP type IA receptor reduced vSMC proliferation and migration in vitro. They hypothesized that BMP4 expression is increased and the BMP pathway is activated to counterbalance the proliferative and chemoattractant effects of other growth factors such as PDGF that are also upregulated in vivo [43]. Given that stimulation with recombinant BMPER protein also induces a contractile vSMC phenotype in vitro and has the tendency to reduce neointima formation in vivo, we suppose that BMPER at least partially upon activating the BMP pathway promotes the contractile vSMC phenotype. Altogether, these findings indicate that a tightly regulated and functional BMP signaling pathway is necessary to keep vSMCs in a contractile, quiescent, and healthy state.

Interestingly, extracellular growth factor modulators are known to regulate different signaling pathways and are rarely pathway-specific [47]. Of note, also some BMP modulators such as the cerberus or connective tissue growth factor (CTGF) have been shown to modulate more than just the BMP pathway. Besides BMPs, CTGF was shown to inhibit BMP signaling on the one hand and to promote TGF-β signaling on the other hand [48]. This circumstance has also been confirmed for BMPER that was shown to interact with low-density lipoprotein receptor-related protein 1 (LRP1) in addition to the BMPs and BMP receptors [49]. Here, we have now add IGFBP4 to the list of BMPER binding partners.

**Figure 8:**
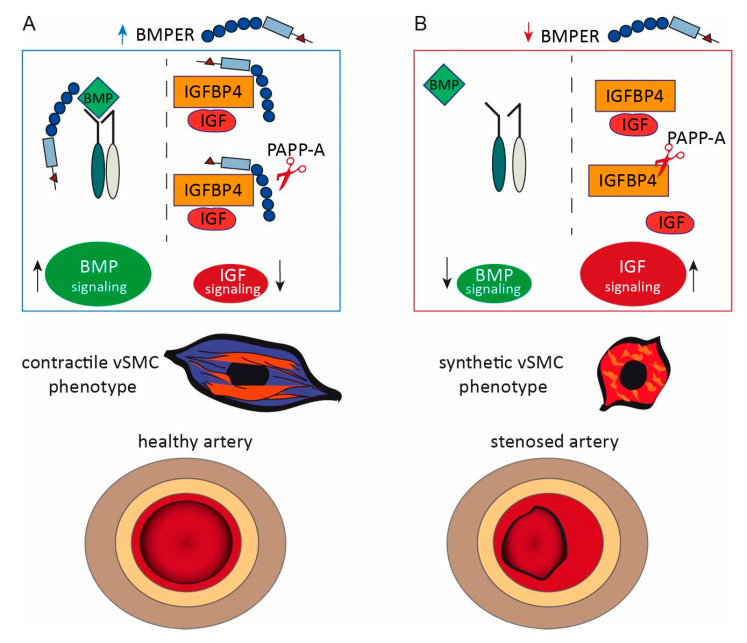
Proposed model of BMPER: interaction with the BMP and IGF signaling pathway in vSMC phenotype modulation and neointima formation. (**A**) If BMPER protein is available, the BMP signaling pathway activity is increased and IGFBP4 is protected from PAPP-A proteolysis. Thus, less IGF is released and the activity of the IGF signaling pathway is reduced. Under these conditions, the contractile vSMC phenotype is supported and neointima formation is reduced. (**B**) In conditions of BMPER loss, BMP signaling pathway activation is decreased. IGFPB4 antagonizes IGF but is cleaved by protease PAPP-A resulting in the release of IGF and the activation of IGF signaling pathway. These conditions contribute to a synthetic vSMC phenotype. An imbalance om the synthetic vSMC phenotype fuels neointima formation after carotid artery injury.

IGFBP4 itself is an extracellular modulator that antagonizes IGFs and is cleaved by the protease PAPP-A. The IGF signaling pathway is characterized to promote the synthetic vSMC phenotype and IGF, and PAPP-A expression is increased in restenosis and as a reaction to vascular injury [36,50]. Several studies have described IGFBP4 as being the major IGFBP produced by vSMCs, and its expression is upregulated after carotid ligation [51,52]. Interestingly, IGFBP4 cleavage by PAPP-A is IGF-dependent, and it is hypothesized that PAPP-A can act as an IGF regulator through the proteolysis of IGFBP4, which ultimately causes neointimal hyperplasia [52,53]. This is underlined by a study with a protease-resistant form of IGFBP4 that was reported to inhibit IGF and neointima expansion in a porcine model of neointimal hyperplasia [54]. We have found that BMPER not only binds to IGFBP4, but also inhibits the proteolytic cleavage of IGFBP4 by PAPP-A in the presence of IGF-1. Therefore, it is tempting to speculate that BMPER, in addition to the BMP pathway, promotes the contractile vSMC phenotype by binding to IGFBP4 and in this way prevents the release of IGF, which itself would trigger the synthetic phenotype (please also refer to scheme in Figure 8).

By definition, BMPER is a matricellular protein because it is a non-structural protein that is located in the ECM and binds to structural ECM components such as heparan sulfate proteoglycans [55]. Furthermore, BMPER has regulatory roles for the BMP and the IGF pathway, and thus, BMPER belongs to the category of matricellular proteins [56]. By modulation of signaling pathway activities in endothelial cells, BMPER mediates anti-inflammatory [23,24,25], and moderate pro-angiogenic [18,38,49] effects, and increases the expression of endothelial nitric oxide synthase (eNOS) [24]. It is well known that eNOS and its product nitric oxide control vasomotor function ad cardiovascular homeostasis and support the differentiated vSMC phenotype [57]. In our present study, we show that BMPER promotes the contractile vSMC phenotype and diminishes neointima formation in the carotid ligation model. In general, BMPER has beneficial effects on both endothelial and vSMCs and we are tempted to call it a “wellness factor” for blood vessels. Of note, there are very few other examples, including interleukin-19, that have anti-inflammatory and at the same time pro-angiogenic effects [58]. Growth factors or cytokines with these properties are of interest in the context of a therapeutic approach to cardiovascular diseases, such as myocardial infarction or ischemic tissues per se, as they could stimulate neovascularization in ischemic tissue while simultaneously attenuating the existing tissue-damaging inflammation. 

Collectively, these data emphasize the extracellular matrix protein BMPER with its rare and valuable characteristics as a future therapeutic agent with high potential. 

## 4. Materials and Methods

### 4.1. Mice

Many large population-based cohort studies report differences in atherosclerotic diseases in different sexes [59]. To limit the potential effects of female hormones, only male mice ranging from 10 to 12 weeks of age were studied. Transgenic B6SJL-Bmper^tm1Emdr^/J mice were originally purchased from the Jackson Laboratory, and a congenic strain on C57/BL6N background was generated (>10 back-crosses). Because BMPER^−/−^ animals die at birth [20], BMPER^+/−^-deficient mice compared to wildtype littermates were used in experiments. For the application of recombinant mouse BMPER protein wildtype C57/BL6N, mice were purchased from Charles River, Sulzfeld, Germany, or from the local stock of the animal facility at the University Medical Center Freiburg, Germany. Mice were housed under specific pathogen-free conditions and had ad libitum access to water and food. Handling and care of animals were approved and in compliance with the guidelines for the care and use of laboratory animals published by the directive 2010/63/EU of the European Parliament. Experimental animal protocols were approved in advance by the Regierungspraesidium Freiburg, Germany.

### 4.2. Mouse Carotid Artery Ligation and Cuff-Induced Neointimal Formation Model 

To induce neointima formation in the mouse carotid artery, we combined the two methods of carotid artery ligation directly under the bifurcation [29] with polyethylene cuff placement below the ligation around the carotid artery [60] (please refer to Figure 1A). Mice were anesthetized with ketamine (Ketaset^®^, Zoetis Deutschland GmbH, Berlin, Germany)/xylazine (Rompun^®^ BAYER, Leverkusen, Germany) (100/10 mg/kg bw, i.p.) in 0.9 saline, followed by preemptive caprofen (Rimadyl, Zoetis Deutschland GmbH, Berlin, Germany (5 mg/kg bw, s.c.)) for post-operative analgesia. Mice were secured in the supine position on a warm heating pad to avoid cooling. The surgical area was shaved and disinfected with Cutasept (Paul Hartmann AG, Heidenheim, Germany). Dexpanthenol eye and nose cream (Bepanthen, BAYER, Leverkusen, Germany) was applied to prevent corneal desiccation. With the scalpel, an approx. 1 cm long incision was made on the right side of the neck. The right common carotid artery (RCCA) was dissected and ligated with 6-0 black braided silk sutures (Look^TM^, Surgical Specialties Corporation, Westwood, MA, USA) near the carotid bifurcation. After ligation, a perivascular polyethylene cuff (0.58 mm inner diameter, 0.964 mm outer diameter, length 5 mm, Becton Dickinson, Franklin Lakes, NJ, USA) was placed around the artery, thereby inducing mechanical stimulation of neointima formation. Finally, the wound was closed with single-button sutures braided with 3-0 Mersilene^®^ (Ethicon, Norderstedt, Germany) suture. The animals were placed in a cage with red light to be warmed and observed until full consciousness was restored and then returned to the colony. To administer recombinant BMPER protein (c = 5 µg/mL recombinant mouse BMPER in 0.9% saline; R&D Systems, Darmstadt, Germany) to C57/BL6N mice, the protein was mixed with 30% Pluronic^®^ gel (Sigma-Aldrich, Schnelldorf, Germany) and applied to the perivascular cuff. The Pluronic gel solidifies at body temperature, forming a gel matrix in the cuff to act as a local drug delivery system in a physiological environment. The control group received the same volume 0.9% saline into the Pluronic gel and cuff. For mRNA expression and (immune-) histochemistry analysis, the injured RCCA and the untreated left common carotid artery (LCCA) were harvested at 14 days or 21 days, respectively.

### 4.3. Histological Examination and Immunostaining

Excised carotid arteries were embedded in Tissue-Tek O.C.T. compound (Fisher Scientific GmbH, Schwerte, Germany) and stored at −20 °C for further histological analysis. Eight-micrometer serial cryostat sections were cut starting from the bifurcation towards the aortic arch. Sections were air-dried, fixed in ice-cold acetone, and subjected to standard hematoxylin and eosin (HE), Movat’s, and Elastica van Gieson (EvG) staining (all Morphisto GmbH, Frankfurt, Germany). To assess morphological changes and to identify the neointimal thickness (represented as ratio of intima/media), neointimal area, and media area, slides were analyzed by a blinded investigator with Zeiss Axioplan2/Axiovision Rel. 4.8 software or Zeiss Axio Imager Z2/ZEN 3.1 blue edition software. Immunofluorescent staining was performed for αSMA-FITC, BMPER, Col3A1, MMP2, and MMP9 compared to respective mouse or rabbit IgG controls (Supplementary Table S1). AlexaFluor555–conjugated secondary antibody was used, and nuclei were stained with DAPI (Sigma-Aldrich). Slides were imaged with Zeiss Axioplan2/Axiovision Rel. 4.8.

Confocal images of human vSMCs stained with anti-αSMA-Cy3 antibody (C6198, clone 1A4, Sigma-Aldrich) were taken by using a ZEISS LSM5 Live DUO high-speed confocal microscope at the Life Imaging Center, ZBSA, Freiburg, Germany.

### 4.4. RNAscope In Situ Hybridization

In situ hybridization with fluorophor–labeled RNA probes was performed by using the RNAscope^TM^ (ACD Biosciences, Newark, NJ, USA) technology, referring to the manufacturer’s instructions. Briefly, carotid arteries were fixed 14 days after carotid ligation with 10% freshly prepared PFA and embedded in O.C.T. Pretreatment with hydrogen peroxide was followed by target retrieval and specific probe hybridization and signal amplification. Afterwards, nuclei were counterstained with DAPI and slides were mounted to proceed with microscopic evaluation. 

### 4.5. Cell Culture 

Human primary pulmonary arterial vascular smooth muscle cells (vSMCs) were purchased and cultured in smooth muscle cell growth medium with 5% fetal bovine serum (FBS) from PELOBiotech (Martinsried, Germany). HEK293A was cultured in DMEM supplemented with 10% FBS (both Gibco^®^). Recombinant proteins (please refer to supplementary data table) were reconstituted according to the manufacturer’s protocol (R&D Systems, Wiesbaden, Germany). For siRNA transfection, cells were seeded the day before and transfected in endothelial basal medium (EBM) containing 0.4% FBS. For recombinant protein or FBS stimulation, cells were seeded in culture medium, and the next day, the cells were synchronized for 24 h in 0.4% FBS EBM before the stimulation started.

### 4.6. Transfection

Silencing of BMPER by siRNA transfection in vSMC was performed as recently described. BMPER siRNAs (B1 and B2) were purchased from Thermo Fisher Scientific, Karlsruhe, Germany. Allstars negative control Alexa Fluor-488 nm was purchased from Qiagen, Hilden, Germany. For transfection, a final concentration of 100 nmol/L siRNA together with Lipofectamine RNAiMAX was used according to the manufacturer’s protocol (Invitrogen^TM^). Transfection efficacy was confirmed by quantitative real-time (q) PCR and Western blot analysis. 

For plasmid transfection in HEK293A cells, FuGENE HD transfection reagent (Promega; Mannheim, Germany) was used. In brief, up to two plasmids were diluted in OptiMEM (Gibco^®^), and FuGENE^®^ HD transfection reagent was added in the ratio of 1:3 (DNA:Fugene). After 15 min incubation time at room temperature, the transfection mixture was added to 80% confluent cell dishes and incubated for 24 h. For a list of plasmid constructs please refer to supplementary Table S1.

### 4.7. Proliferation Assay 

Proliferation was assessed using a colorimetric BrdU-incorporation ELISA (Roche, Basel, Switzerland). In brief, after 24 h siRNA transfection or stimulation with recombinant proteins, cells were cultured in fresh BrdU-containing 0.4% FBS/EBM medium for another 24 h. The colorimetric ELISA for BrdU quantification was performed following the manufacturer’s instructions.

### 4.8. Migration Assay

Cell migration assay was performed as previously described [18]. In brief, vSMCs pre-stimulated with indicated recombinant proteins or transfected with siRNAs for 48 h were labeled with 10 µM CFDA-SE (Life Technologies), harvested by centrifugation, resuspended in migration medium (RPMI with 0.5% FBS, 0.1% BSA), counted, and placed in the upper chamber of a modified Boyden chamber (1 × 10^5^ cells per HTS FluoroBlok 24-well chamber; pore size 8 µm; BD Biosciences, Heidelberg, Germany). The chambers were placed in 24-well culture dishes containing migration medium and indicated recombinant proteins. After incubation for 4 h at 37 °C, 5% CO_2_, the cells were fixed with 4% PFA and migrated cells were counted manually in 5 random microscopic fields using an Axiovert fluorescence microscope.

### 4.9. Collagen-Gel Contraction Assay

After 6 h siRNA transfection, 5 × 10^4^ vSMCs were mixed with 250 µL of freshly prepared collagen gel solution (Cultrex Rat Collagen I, R&D Systems, 0.1% acetic acid, 1M sodium hydroxide solution, HEPES buffer (Gibco), 10× Medium 199 (Sigma-Aldrich)), seeded in 48 well plates and incubated for 30 min at 37 °C in a cell incubator to induce gelation. Afterwards, 250 µL 0.4% FBS/EBM was added for 48 h, before the gels were lifted off the bottom of the wells and allowed to float freely. Twenty-four hours later gels were stained with phenol red to increase the contrast, imaged, and gel-size-quantified with a digital imaging system (ChemiDOC XRS (Bio-Rad)).

### 4.10. Cell Viability Assay

Cell viability was assessed by using the CellTiter-Fluor™ cell viability kit from Promega, Mannheim, Germany. CellTiter-Fluor™ measures a conserved and constitutive protease activity within live cells and therefore serves as a marker of cell viability. Briefly, 7000 vSMCs per 96 wells were seeded in 5% SMC medium before stimulation with recombinant proteins or siRNA transfection in 0.4% EBM was performed. Cell viability assay was performed following the manufacturer’s instructions. The resulting fluorescence signal of each well was measured by using the Gemini fluorescence microplate reader and SoftMaxPro software (Molecular Devices) set to 380 nmEx/505 nmEm.

### 4.11. Cell Apoptosis Assay

Cell apoptosis was assessed by using the luminescence Caspase-Glo^®^3/7 assay kit from Promega. Caspase-Glo^®^3/7 is a proluminescent caspase-3/7 substrate, which is cleaved to release aminoluciferin, a substrate of luciferase used in the production of light. Cells were treated as described for cell viability assy. After 48 h, cell apoptosis assay was performed following the manufacturer’s instructions. The resulting luminescence signal of each well was measured by using the GloMax^®^ 96-microplate luminometer (Promega).

### 4.12. In Situ Proximity Ligation Assay (PLA)

To detect direct BMPER–IGFBP4 physical interaction, either vSMCs were grown on 12 well glass coverslips (ibidi, Graefelfing, Germany) or carotid tissue sections were fixed with acetone for 20 min at −20 °C and washed 3 times in PBS. Subsequently, the Duolink^®^ PLA assay was performed following the manufacturer’s instructions (Sigma-Aldrich). In brief, rabbit anti-Igfbp4 antibody and rat anti-Bmper antibody were incubated overnight at 4 °C. Afterwards, anti-rabbit and anti-mouse oligonucleotides labeled secondary antibodies (PLA probes) were incubated, followed by a ligase and polymerase reaction to amplify the signal. DAPI was used to stain the nuclei before photographs were taken by Zeiss Axio Imager Z2 with ApoTome/ZEN 3.1 blue edition software.

### 4.13. RNA Extraction and Reverse Transcription

Total RNA from mouse carotid arteries was extracted using the TriPure isolation method according to the manufacturer’s protocol followed by DNase I treatment (Sigma-Aldrich). Reverse transcriptions were performed with iScript cDNA-Kit, applying 1 µg RNA following the manufacturer’s protocol (Bio-Rad, Munich, Germany).

### 4.14. Quantitative Real-Time PCR

Quantitative real-time PCR was performed using IQ SybrGreen 2 × Supermix (BioRad) or TaqMan^®^ gene expression master mix and assays (Thermo Fisher Scientific) that were analyzed with the iCycler real-time PCR detection system or CFX96 touch real-time PCR detection system (Bio-Rad). Quantification was performed using MyiQ lightcycler or CFX manager version 3.1 software (Bio-Rad). Differences in gene expression were calculated using the ΔΔCT method [61]. The housekeeping gene human RNA polymerase II (hRP) was used for internal normalization. Primers were purchased from Eurofins MWG Operon, Ebersberg, Germany. For primer sequences and TaqMan kits, please refer to Supplementary Table S1.

### 4.15. Western Blot Analysis

Western blot analysis was performed as previously described. Primary antibodies were incubated overnight at 4 °C and secondary antibodies at room temperature for 1 h in 3% non-fat dried milk/TBST. Visualization was performed by an ECL system (GE Healthcare Europe GmbH, Freiburg, Germany) and a digital imaging system (ChemiDOC XRS (Bio-Rad)). For quantification of protein band intensities, Image Lab (Bio-Rad, Munich, Germany) was used and expression was normalized to Gapdh loading control.

### 4.16. IGFBP4 Protease Assay

Twenty-four hours after plasmid transfection, the supernatants were harvested by centrifugation, and IGF1 (c = 25 ng/mL) was pre-incubated at 37 °C for 15 min at 37 °C in an incubator before PAPP-A (c = 150 ng/mL) was added and incubated for another 2 h at 37 °C. Afterwards, supernatants were transferred for concentration on centrifuge tubes with Amicon^®^ Ultra-4 10 K centrifugal filter units (Millipore) and 50 μL protein concentrate was subjected to Western blot analysis. 

### 4.17. Immunoprecipitation (IP)

Co-immunoprecipitation was used to demonstrate the potential protein–protein interaction of BMPER and IGFBP4. Twenty-four hours after plasmid transfection, HEK293A cells were mechanically detached in PBS w/o and centrifuged at 500× *g* for 5 min at 4 °C and the pellet was resuspended in 500 μL non-denaturing IP lysis buffer. Cell disruption was then performed by repeated freezing in liquid nitrogen. Following lysis, lysates were centrifuged at 4 °C for 10 min at 12,000 g, and aliquots were set aside for direct blot analysis (input). For the IP 5 μg of the specific antibody was added to the cell lysate and incubated overnight at 4 °C rotating. The next day, immunocomplexes were precipitated at 4 °C for 1 h with protein G PLUS-agarose beads (Santa Cruz, Heidelberg, Germany) and subsequently washed three times with 1 mL IP buffer for 10 min and centrifugation at 2000× *g* for 5 min at 4 °C. Proteins were eluted with 6× Laemmli sample buffer and heated for 5 min at 95 °C before Western blot analysis was performed.

### 4.18. Surface Plasmon Resonance (SPR)

SPR experiments were performed as described previously (using a BIAcore 2000 system (BIAcore AB, Uppsala, Sweden)). BMP-4 and IGFBP-4 were immobilized at 430 and 360 RUs, respectively, to a CM5 sensor chip using the amine coupling kit following the manufacturer’s instructions (Cytiva, Uppsala, Sweden). Interaction studies were performed by injecting 0–320 nM recombinant BMPER and IGFBP-4 in HBS-EP buffer (0.01 M HEPES, pH 7.4, 0.15 M NaCl, 3 mM EDTA, and 0.005% (*v*/*v*) surfactant P20) (Cytiva). For competition assays, BMPER was preincubated for 30 min at a constant concentration of 0.75 nM with 0–6 nM IGFBP-4 prior to injection. The surface was regenerated with a pulse of 10 mM glycine, pH 1.5. Kinetic constants were calculated by nonlinear fitting (1:1 interaction model with mass transfer) to the association and dissociation curves according to the manufacturer’s instructions (BIAevaluation version 3.0 software). Apparent equilibrium dissociation constants (KD values) were then calculated as the ratio of kd/ka.

### 4.19. Statistical Analysis

Statistical analysis was performed using GraphPad Prism 5.0, La Jolla, USA. Data are presented as mean ± SEM, and comparisons were calculated by Student’s *t*-test (2-sided, unpaired). All experiments were repeated at least three times in triplicates. One-way ANOVA was used for multiple comparisons of >2 groups. The Bonferroni post-test for multiple comparisons was used if the *p*-value for the overall ANOVA comparison was statistically significant. Results were considered statistically significant for *p* < 0.05.

## Data Availability

The data presented in this study are available in this article.

## Supplementary Materials

The supporting information can be downloaded at https://www.mdpi.com/article/10.3390/ijms24054950/s1.
