# Supplementary material for: BMPER Improves Vascular Remodeling and the Contractile Vascular SMC Phenotype
Source: Int J Mol Sci. 2023 Mar 3;24(5):4950. doi: 10.3390/ijms24054950 (PMC10002482; doi:10.3390/ijms24054950)
Supplement: Supplementary file 1 [file ijms-24-04950-s001.zip › ijms-2227515-supplementary.pdf]

International Journal of
Molecular Sciences

MDPI

Supplementary Materials

Figures

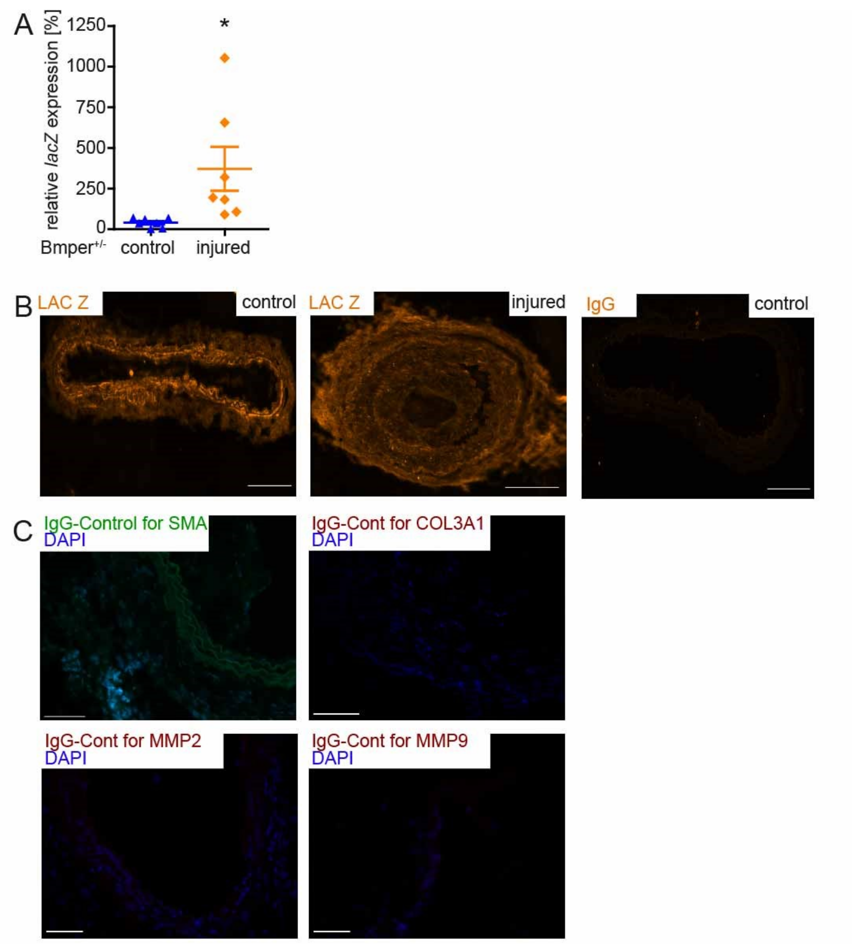

Figure S1. Mouse carotid artery ligation and cuff-induced neointimal formation model causes changes in bone morphogenetic protein-binding endothelial regulator (BMPER) promoter-driven *lacZ* expression**. A**, Quantitative real-time PCR analysis of *lacZ* mRNA expression levels 14 days after carotid injury in C57BL/6N_Bmper$^{+/-}$ mice. RNA polymerase II serves as internal control. Data represent mean values with SEM; n=7 per group;**P*=as indicated vs control. **B,** After 21 days right common carotid artery (RCCA) and left CCA (LCCA) were harvested and frozen in embedding media before cryosections were prepared. Representative micrographs of *Bmper* promoter-driven LAC Z immunostaining (orange), and 4′,6-diamidino-2-phenylindole (DAPI) to visualize nuclei (blue). Scale bar: 100 µm. **C**, Representative pictures of IgG control staining for SMA, COL3A1, MMP2 and MMP corresponding to the main Figure 2 of the manuscript. Scale bar 50 µm.

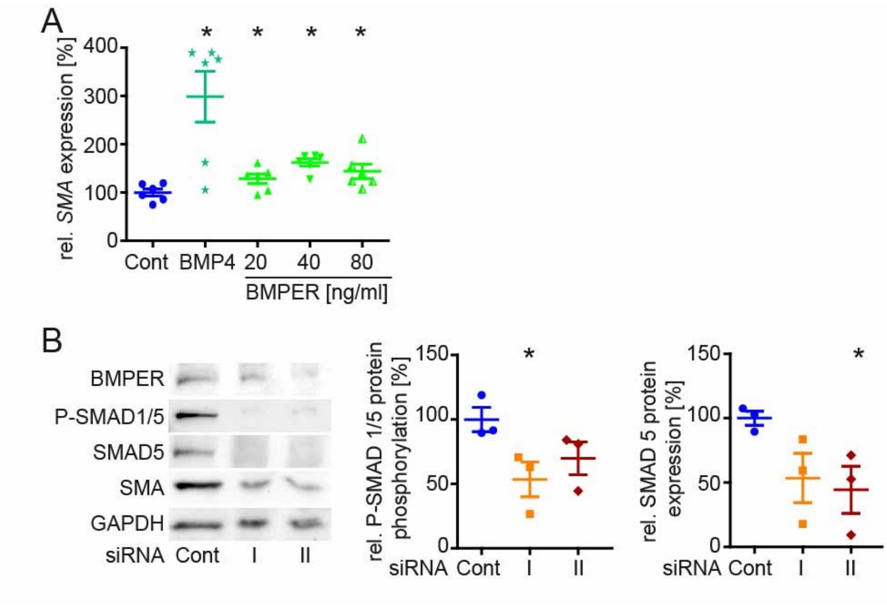

Figure S2. BMPER and classical BMP signaling pathway activation**. A,** After initial serum starvation in 0.4% EBM for 24 hours, vSMCs were stimulated with indicated recombinant proteins before expression analysis of SMA by qRT-PCR was performed. Human RNA polymerase II serves as internal control. Data are mean±SEM; n=6; *P<0.048 vs control. **B**, Human vSMCs were silenced for BMPER with either of two specific small interfering RNAs (siRNAs; BI and BII) or transfected with control siRNA (Cont). Western blot analysis of BMPER, P-SMAD1/5, SMAD5, and SMA was performed 72 hours post siRNA transfection. Glyceraldehyde 3-phosphate dehydrogenase (GAPDH) serves as loading control. Data represent mean±SEM; n=3; *P<0.048 vs control.

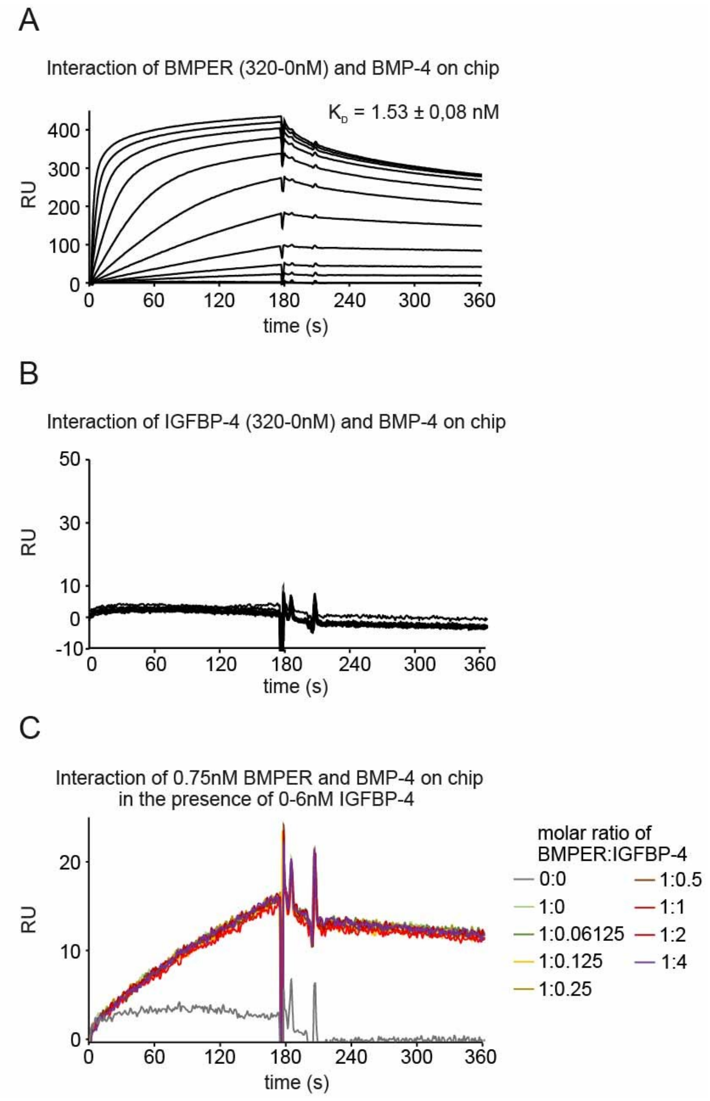

Figure S3. Surface plasmon resonance (SPR) analysis of BMPER, IGFBP4 and BMP4 interaction**. A,** Representative surface plasmon resonance (SPR) sensorgram showing the interaction of immobilized BMP4 with soluble BMPER (KD of 1.53±0.08 nM). 0 – 320 nM BMPER diluted in HBS-EP buffer was injected. **B,** Representative SPR sensorgram showing no interaction of immobilized BMP4 with soluble IGFBP4. 0 – 320 nM IGFBP4 diluted in HBS-EP buffer was injected. **C,** Competition assay. Representative SPR sensorgram showing the interaction of immobilized BMP4 with soluble BMPER (0.75 nM) and IGFBP4. 0 – 6 nM IGFBP4 diluted in HBS-EP buffer was injected. IGFBP-4 does not interfere with the BMPER-BMP-4 interaction.

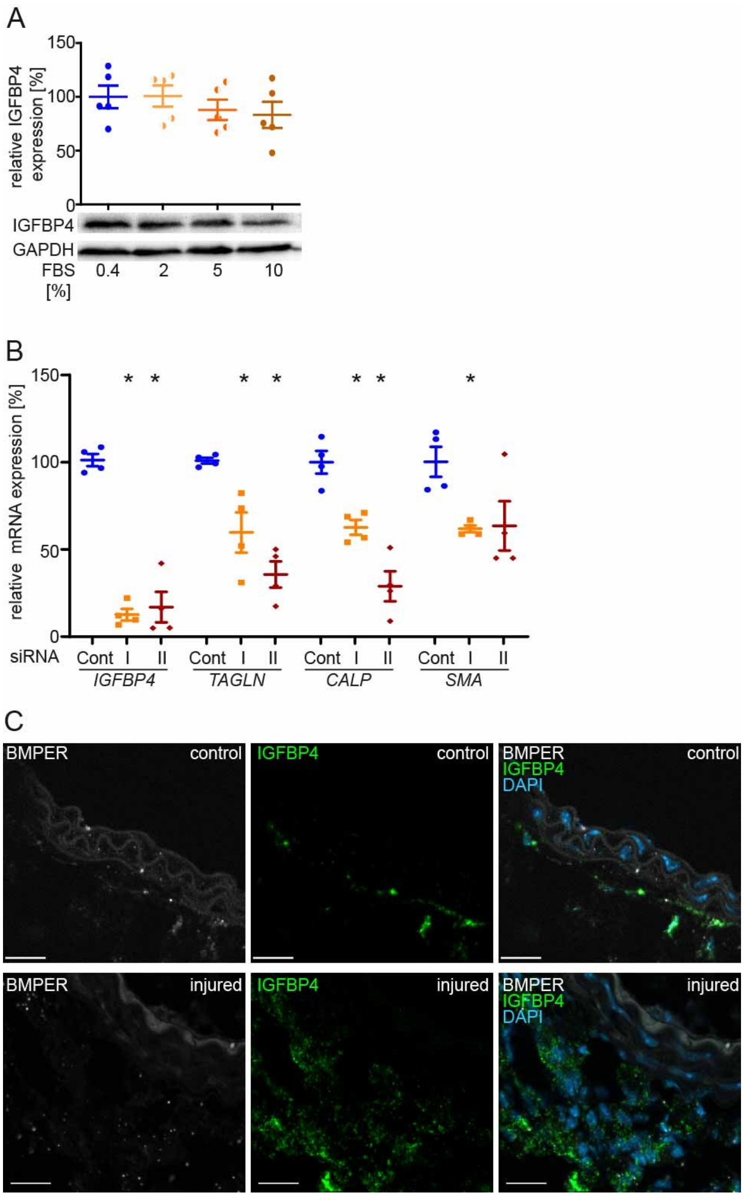

Figure S4. IGFP4 regulation by serum, effect of IGFBP4 silencing on contractile vSMC markers and RNAscope *in situ* hybridisation for BMPER and IGFBP4 mRNA expression of sections from carotid artery. **A**, Western blot analysis of IGFBP4 protein expression was performed from vSMCs at 48 hours of stimulation with different FBS concentrations. GAPDH serves as loading control. Data represent mean±SEM; n=5. **B,** Human vSMCs were silenced for IGFBP4 with either of two specific small interfering RNAs (siRNAs; I and II) or transfected with control siRNA (Cont). Quantitative real-time PCR analysis of *IGFBP4*, *transgelin* (*TAGLN*), *calponin (CALP), SMA* and *myosin heavy chain 11* (*MYH11*) mRNA expression levels 72 hours post-transfection. Human RNA polymerase II serves as internal control. Data are mean±SEM; n=4;*P<0.0123 vs control. **C,** Representative micrographs of RNAscope *in situ* hybridization of *Bmper* (white) and *Igfbp4* (green) mRNA expression in untreated (top) and injured (bottom) carotid artery 14 days post operation. Nuclei are visualized by 4′,6-diamidino-2-phenylindole (DAPI) (blue). Scale bar: 20 µm.

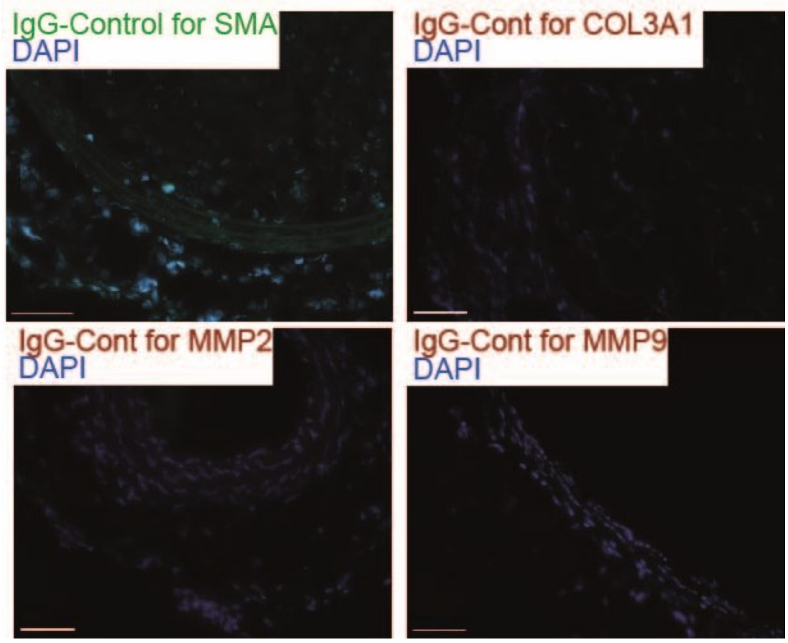

Figure S5. Representative pictures of cross-sections stained for the IgG controls for SMA (green), COL3A1 (red), MMP2 (red) and MMP9 (red) for the corresponding figure 7. DAPI staining to visualize the nuclei appears in blue. Scale bar: 50 µm.

Table S1. Primer sequences, Taqman assays, siRNAs, antibodies.

		**Primer**	
**Gene**	**Species**	**Forward**	**Reverse**
Bmp4	human	5′-CACGAAGAACATCTGGAGAAC-3′	5′-CCCTTGAGGTAACGATCAGCT-3′
Bmper	human	5′- CTTGTGTTCTACGCCAGTGCC-3′	5′-GGACAGGAGAGAATGGGACAGAC-3′
Bmper	mouse	5′-GGTGTCTGTAACAGTGATGA-3′	5′-CAGTGCAGATAGGACAGCATC-3′
Calp	human	5′-AGCTAAGAGAAGGGCGGAAC-3′	5′-CATCTGCAGGCTGACATTGA-3′
Col1a1	mouse	5′-CTGGCGGTTCAGGTCCAA-3′	5′-TTCCAGGCAATCCACGAG-3′
Col3a1	mouse	5′-CAAGGGTGATCGTGGTGAAAA-3′	5′-CCAGGGAATCCTCGATGTCCT-3′
Fbn1	mouse	5′-CATTCCTGTGGGGATGGATTC-3′	5′-TACGTGCAAGCACACCGATTT-3′
LacZ	E.coli	5′-TATTGGCTTCATCCACCACA-3′	5′-TTGAAAATGGTCTGCTGCTG-3′
Myh11	human	5′-CATCTACTCGGAGAAGATCGTCG-3′	5′-CGCCTGTGCATAGAATGGACT-3′
Mmp2	mouse	5′-GCTGGGAGCATGGCGATGGATAC-3′	5′-GGAAGCGGAATGGAAACTTC-3′
RplI	human/ mouse	5′-GCACCACGTCCAATGACAT-3′	5′-GTGCGGCTGCTTCCATAA-3′
Sma	human	5′-GCGTGGCTATTCCTTCGTTA-3′	5′-ATGAAGGATGGCTGGAACAG-3′
Sma	mouse	5′-CCCAGACATCAGGGAGTAATG-3′	5′-TCTATCGGATACTTCAGCGTC-3′
Tagln	human	5′-AACAGCCTGTACCCTGATGG-3′	5′-CGGTAGTGCCCATCATTCTT-3′

		**siRNA**	
**Gene**	**Species**	**Forward**	**Reverse**
Bmper_1	human	5′-GCACCUUAGUCACAUACCCTT-3′	5′-GGGUAUGUGACUAAGGUGCTG-3′
Bmper_2	human	5′-GCUGCCUCUUUCGAAGUGATT-3′	5′-UCACUUCGAAAGAGGCAGCTC-3′

**TaqMan Assay**			
**Assay name**	**Assay ID**	**Cat. No.**	**Company**
Mm_Mmp9	Mm00442991_m1	4331182	Thermo Fisher Scientific
Mm_ Actb	Mm00607939_s1	4352933E	Thermo Fisher Scientific

Recombinant Proteins			
**Protein**	**Species**	**Cat. No.**	**Company**
BMP4	human	314-BP	R&D Systems
BMPER	human	1956-CV	R&D Systems
Bmper	mouse	2299-CV	R&D Systems
Chordin	mouse	758-CN	R&D Systems
IGF-1	human	291-G1	R&D Systems
IGFBP-4	human	804-GB	R&D Systems
Noggin	human	6057-NG	R&D Systems
PAPP-A	human	2487-ZNF	R&D Systems
PDGF	human	220-BB	R&D Systems
TGFβ1	human	240-B	R&D Systems

Primary antibodies					
**Against**	**Host species**	**Clone ID**	**Cat. No.**	**Company**	**(label)**
Bmper	rabbit	polyclonal	ab739000	Abcam	
Bmper	rat	#355304	MAB1956	R&D Systems	
β-galctosidase (LacZ)	chicken	polyclonal	Ab9361	Abcam	
Col3a1	rabbit	polyclonal	Ab7778	Abcam	
Igfbp4	rabbit	polyclonal	Sc-13092	Santa Cruz	
Myh11	rabbit	monoclonal	Ab124679	Abcam	
c-myc	mouse	9E10.3	DLN-07722	Dianova	
αSMA	mouse	1A4	A5228	Sigma-Aldrich	
αSMA	mouse	1A4	F3777	Sigma-Aldrich	FITC
αSMA	mouse	1A4	C6198	Sigma-Aldrich	Cy3
V5	mouse	monoclonal	R960-25	Thermo Fisher Scientific	
Mmp2	rabbit	polyclonal	ab97779	Abcam	
Mmp9	rabbit	polyclonal	ab38898	Abcam	
Gapdh	rabbit	polyclonal	E1C604-1	EnoGene	
Vimentin	rabbit	D21H3	5741	Cell Signaling	
IgG control	rabbit	polyclonal	X0936	DakoCytomation	
IgG control	rat	IgG2a	553930	BD Pharmingen	PE
IgG control	mouse	IgG2a	553929	BD Pharmingen	FITC
IgG control	chicken	polyclonal	003-000-003	Jackson	

Secondary antibodies

Against	Host species	Clone ID	Cat. No.	Company	(label)
rabbit	donkey	polyclonal	A-31572	Thermo Fisher Scientific	AlexaFluor555
chicken	goat	polyclonal	**A-11039**	Thermo Fisher Scientific	Alexa488
Mouse	donkey	polyclonal	A90-137D2	Bethyl	Dylight488
rat	goat	polyclonal	**A-21434**	Thermo Fisher Scientific	Alexa Fluor 555
**Plasmids**	**Host species**		**Tag**	**Source**	
pcDNA3	N/A		N/A	Invitrogen, Karlsruhe, Germany	
pcDNA3_hBMPER	human		N/A	Jennifer Esser, Freiburg, Germany	
pcDNA4	N/A		V5, 6xHis	Invitrogen, Karlsruhe, Germany	

pcDNA4_mIgfbp4	mouse	V5, 6xHis	Dr. Ichiro Shiojima, Chiba University, Japan
pSecTag2A	N/A	Myc, 6xHis	Invitrogen, Karlsruhe, Germany
pSecTag2A_mBMPER	mouse	Myc, 6xHis	Martin Moser, Freiburg, Germany
